# Comparative Studies of the Pyrolytic and Kinetic Characteristics of Maize Straw and the Seaweed *Ulva pertusa*

**DOI:** 10.1371/journal.pone.0012641

**Published:** 2010-09-10

**Authors:** Naihao Ye, Demao Li, Limei Chen, Xiaowen Zhang, Dong Xu

**Affiliations:** 1 Key Laboratory for Sustainable Utilization of Marine Fishery Resources, Yellow Sea Fisheries Research Institute, Chinese Academy of Fishery Sciences, Qingdao, China; 2 Food Science Department, College of Agriculture, Liaocheng University, Liaocheng, China; University of Sydney, Australia

## Abstract

Seaweed has attracted considerable attention as a potential biofuel feedstock. The pyrolytic and kinetic characteristics of maize straw and the seaweed *Ulva pertusa* were studied and compared using heating rates of 10, 30 and 50°C min^−1^ under an inert atmosphere. The activation energy, and pre-exponential factors were calculated by the Flynn-Wall-Ozawa (FWO), Kissinger-Akahira-Sunose (KAS) and Popescu methods. The kinetic mechanism was deduced by the Popescu method. The results indicate that there are three stages to the pyrolysis; dehydration, primary devolatilization and residual decomposition. There were significant differences in average activation energy, thermal stability, final residuals and reaction rates between the two materials. The primary devolatilization stage of *U. pertusa* can be described by the Avramic-Erofeev equation (n = 3), whereas that of maize straw can be described by the Mampel Power Law (n = 2). The average activation energy of maize straw and *U. pertusa* were 153.0 and 148.7 KJ mol^−1^, respectively. The pyrolysis process of *U.pertusa* would be easier than maize straw. And co-firing of the two biomass may be require less external heat input and improve process stability. There were minor kinetic compensation effects between the pre-exponential factors and the activation energy.

## Introduction

In recent years, marine origin biomass such as seaweed, has attracted considerable attention as a potential biofuel feedstock. Seaweeds are an important component in marine ecosystems providing an important and unique ecological function. As a potential biofuel feedstock, macroalgae seaweeds have a number of desirable features, such as fast growth, high biomass conversion rate, short growth cycle, ease of handling and the potential for zero net CO_2_ emissions.

Pyrolysis can be used to harvest the energy contained in macroalgae. The technique has been previously proposed and the pyrolytic characteristics of macroalgae have been examined [1]–[5]. However, the product yield from pyrolysis is still very low and the resulting bio-oils have many complex components which can be corrosive or hygroscopic [1]. Thus, to produce a practical process route further study of the pyrolytic characteristics of macro-algae is required.

*Ulva pertusa* is native to China and exhibits fast growth and a high reproductive capacity. The alga differs from higher plants, by having a cell wall comprises of two layers, the inner layer is composed of cellulose and the external layer is composed of pectin which in turn consists of D-galactose, L-alabinose, D-xylose and an L-rhamnose complex on the cell surface [6]. *U. pertusa* is a green algae. In terms of cell wall construction and biochemical elements, these green algae most closely resemble higher plants.

Chinese agriculture is producing huge amount of maize straw as a by-product [7]. Maize straw can be used for pyrolytic oil production using recently developed technologies such as fast pyrolysis [8].

In this study, maize straw is chosen as a representative of terrestrial crops composed of hemicelluloses, cellulose and lignin, to compare to *U. pertusa* in terms of pyrolytic and kinetic characteristics. The average activation energy, pre-exponential factors, and reaction orders associated with pyrolysis were calculated to facilitate the efficient design, operation, and modeling of pyrolytic and related thermo-chemical conversion systems for both algae and higher plants.

## Results and Discussion

### Characteristics of the thermal degradation process

**Figure 1:**
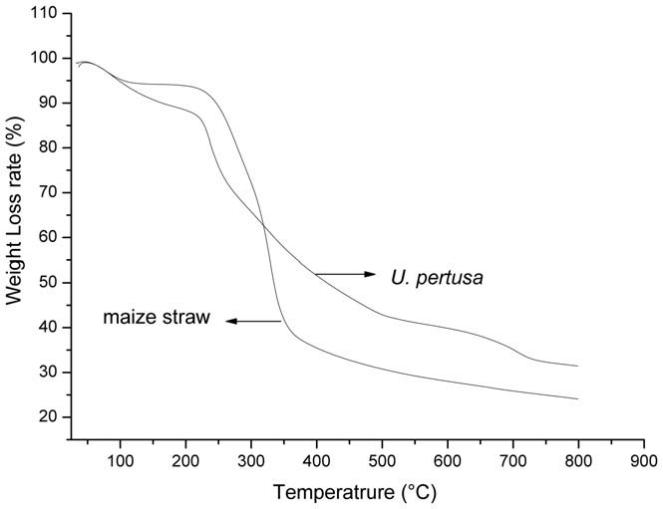
The TG curves of maize straw and *U. pertusa* at different heating rates of 10°C/min.

**Figure 2:**
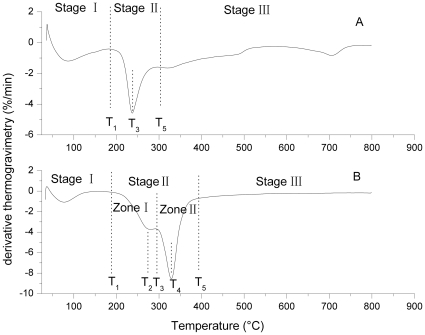
The DTG curves of *U. pertusa* (A) and maize straw (B) at different heating rates of 10°C/min with the characteristic temperature zone.

As previous studies [1]–[2], thermogravimetric (TG) (Fig. 1) and differential thermogravimetric (DTG) (Fig. 2) curves of maize straw and *U. pertusa* indicated that there are three stages in the pyrolytic process. The first stage (I) occurred as the temperature increased from ambient to T_1_, while the second stage (II) occurred as the temperature increased from T_1_ to T_5_. However, the samples revealed large differences in degradation behavior during stage II. Stage II was composed of two zones for maize straw, with zone I occurring as the temperature increased from T_1_ to T_3_ with a maximum weight loss point at T_2_ and zone II occurring as the temperature increased from T_3_ to T_5_ with a maximum weight loss point at T_4_. For *U. pertusa*, there was only one zone in stage II, which occurred as the temperature increased from T_1_ to T_5_ with a maximum weight loss point at T_3_. The third stage (III) occurred as the temperature increased from T_5_ to 800°C. The characteristic temperatures are shown in Table 1 and Fig. 2.

**Table 1:** Temperature characteristics associated with the pyrolysis process.

Samples	Heating rate (°C/min)	Temperature (°C)				
		T_1_	T_2_	T_3_	T_4_	T_5_
Maize straw	10	200	280	291.6	329.1	373
	30	220	296.9	314.6	349.3	400
	50	240	300.5	324.6	357.6	408
*pertusa*	10	182.3		237.6		280
	30	199.3		251.4		305
	50	210		257.9		316

Fu et al. (2008) [9] indicated that the maize straw showed a DTG curve with a single peak and a shoulder. However, Muller-Hagedorn et al. (2007) [10] found that the two maize samples in their studies showed only one wide DTG curve peak. Normally, DTG curves from biomass exhibit a peak at high temperatures that is mainly due to the pyrolysis of the cellulose and a shoulder at lower temperatures that can be attributed to the pyrolysis of the hemicelluloses [10]. A previous study of green algae by our research group [1] showed DTG curves with one wide peak for *Enteromorpha prolifera*, as well as *U. pertusa*.

During stage I, cellular water and the external water bound by surface tension are lost. Stage II is the devolatilization stage, during which the main pyrolytic process occurs. In this stage, various volatile components are gradually released, resulting in a large weight loss and formation of the main pyrolytic products. Stage II occurred over a temperature range of 200–408°C for maize straw and 182.3–316°C for *U. spertusa*. The onset of decomposition occurs at a lower temperature for *U. pertusa* than for maize straw. This may be caused by the low polymerization of the polysaccharides and the presence of inorganic salts in *U. pertusa* [2], [5]. During the third stage, the residue slowly decomposed, resulting in the formation of a loose porous product.

The weight loss is seen in the three samples during stage I (Table 2) were primarily due to the loss of moisture and were similar to the moisture content values reported in Table 3. The amount of the final residue of maize straw at 800°C was lower than *U. pertusa*. In addition, the instantaneous maximum reaction rate for maize straw was higher than *U. pertusa*. For maize straw, the instantaneous maximum reaction rate occurred in zone II of stage II.

**Table 2:** Weight loss and average reaction rate at different stages.

Stage			Maize straw			*Ulva pertusa*		
			Heating rate (°C/min)			Heating rate (°C/min)		
			10	30	50	10	30	50
I	WL^a^(%)		5.8	4.3	6.6	11.16	11.13	13
	AR^b^ (%/min)		0.4	1.1	2.0	0.7	1.9	3.4
II	Z I^c^	WL (%)	18.9	25.0	22.7	19.9	23.6	24.6
		AR (%/min)	1.4	5.1	9.4	2.2	7.2	12.3
	Z II^d^	WL (%)	37.8	36.2	35.7	-	-	-
		AR (%/min)	9.6	14.5	17.8	-	-	-
	IMR^e^ (%/min)		8.5	25.3	44.12	4.6	15.5	28.8
III	WL (%)		29.6	25.5	10.7	37.6	35.77	33.8
	AR (%/min)		0.6	0.8	1.4	0.7	2.2	3.5
Final residue at 800°C (%)			24.1	23.7	24.4	31.4	29.5	28.6

a Weight loss;

b Average reaction rate;

c Zone I;

d Zone II;

e The instantaneous maximum reaction rate;.

**Table 3:** Proximate analysis and chemical content of the samples.

Proximate analysis (received basis, wt%)	Maize straw	*Ulva pertusa*
Moisture (M*_ar_*)	6.6	8.0
Ash (A*_ar_*)	5.7	19.6
Volatile matter (V*_ar_*)	78.0	59.3
Fixed carbon (FC*_ar_*)	9.7	13.1
Qad, net (MJ/kg)	16.9	11.5

Heating rate has significant effect on the pyrolysis of maize straw and *U. pertusa*. As the heating rate increased, the initial pyrolytic temperature, the average reaction rate and the temperature at which the maximum weight loss occurred, all increased (Tables 1 and 2).

**Figure 3:**
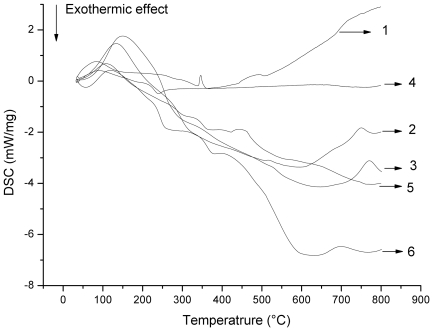
The DSC curves of the samples at different temperature. 1 is the DSC curve of maize straw at heating rate of 10°C/min; 2 is the DSC curve of maize straw at heating rate of 30°C/min; 3 is the DSC curve of maize straw at heating rate of 50°C/min; 4 is the DSC curve of *U. pertusa* at heating rate of 10°C/min; 5 is the DSC curve of *U. pertusa* at heating rate of 30°C/min; 6 is the DSC curve of *U. pertusa* at heating rate of 50°C/min.

As the heating rate increased, the reaction exotherm also increased (Fig. 3). There was an endothermic peak during stage I that corresponded with moisture evaporation. As the temperature increased, an exothermic effect appeared during stage II and exothermic peaks were observed at 5–15°C after the maximum weight loss point. These findings indicate that the devolatilization stage (stage II) produced heat. Moreover, as the temperature continued to rise, there were differences among the differential scanning calorimetry (DSC) curves produced using different heating rates. Specifically, there was an endothermic effect during the maize straw stage III. There was also an exothermic effect during stage III when heating rates of 50°C/min were used for *U. pertusa*.

**Figure 4:**
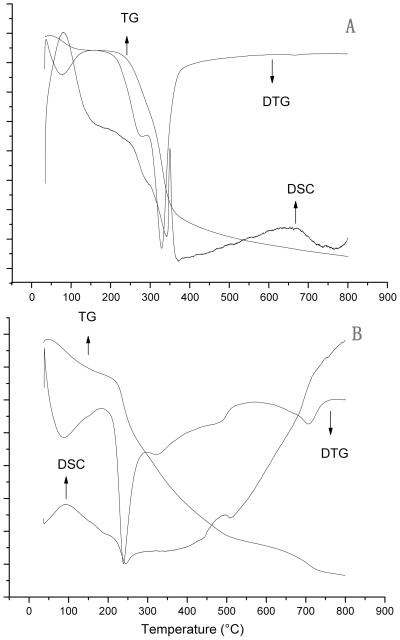
TG-DTG-DSC curves of maize straw (A) and *U. pertusa* (B).

An endothermic maximum peak corresponding to the maximum weight loss rate peak appeared on the maize straw DSC curve (Fig. 4A). However, the highest exothermic peak appeared on the DSC curve of *U. pertusa* (Fig. 4B). This suggests that the pyrolysis of *U. pertusa* is mainly an exothermic process. Generally speaking, an endotherm can be related to depolymerization and volatilization processes, whereas an exotherm is due to charring [3], [11]. This may be caused by the low depolymerization energy requirement and inorganic salts present in *U. pertusa* assisting in robust char formation and exothermic effects [2], [11].

### Kinetic analysis of the pyrolysis process

#### Popescu method for determining the kinetic mechanism

Different conversion rates in stage II at different heating rates and temperatures were chosen to determine the mechanism function (Table 4). Forty-one typical mechanisms [12] were analyzed using the Popescu method. Table 4 shows correlation coefficients (R) and standard deviations (SD).

**Table 4:** The linear fitting results of kinetic mechanism function of the samples.

Sample	Function No.	Temperature (°C)	R	SD
Maize straw	27	250	0.9975	9.56E-04
	Mampel Power	300	0.9977	0.0152
	n^a^ = 2	350	0.9998	0.1543
*Ulva pertusa*	19	215	0.9954	0.0139
	Amirami-Erofeev function	235	0.9911	0.0251
	N = 3	255	0.9970	0.0203
		275	0.9948	0.0188

a Reaction order.

As shown in Table 4, function 19 (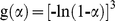) is the best fit function for *U. pertusa* (n = 3), while function 27 (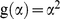) is the best fit function for maize straw (n = 2). For *U. pertusa*, the most probable mechanism may be interpreted as random nucleation and nuclei growth. At first, the decomposition reaction occurred on partional point of solid phase and activation center generated randomly. Thereafter, partial activation center produce decomposition product or inactivated, and partial activation center contnuning to grow followed by newly formed activation center. For maize straw, the most probable mechanism was nucleation.

Wang et al. [3] and Li et al.[2] found that random nucleation and growth was predominant during the main pyrolysis of algae, which is described by the Amirami-Erofeev function. We obtained similar results (n = 3) to the mechanism proposed for *L. japonica* and *S. pallidum* [2].

Maize straw was mainly composed by lignocellulosic material (approximately for 80%). However, *Ulva pertusa* were mainly composed of protein, polysaccharides and little cellulose. Moreover, large amount of inorganic salts in seaweeds may be the other reaons which can induce the heterogeneous nucleation of the volatile [3]. The different mechanisms of maize straw and *U. pertusa* may be the result of differing sample compositions and the physical configuration of the powder.

#### Calculation of the activation energy and pre-exponential factors

The activation energy and lnA of maize straw and *U. pertusa* are listed in table 5. The results have confidence values ranging from 0.9179 to 0.99999. Therefore, the activation energy calculated by the Popescu method, FWO and the KAS methods are valid. However, the activation energy revealed fluctuations related to conversion rates. This may be ascribed to the complex composition of the samples and the complex reactions that occur during pyrolysis. For maize straw, the rank order of the activation energies calculated by the different methods are Popescu method, FWO method and KAS method. However, for *U. pertusa*, the order is FWO method, KAS method and Popescu method. It also indicates that the activation energy calculated by the FWO method is always higher than from the KAS method. The average activation energy of *U. pertusa* (148.7 KJ/mol) was lower than maize straw (153.0 KJ/mol), indicating that *U.pertusa* caught fire easily than maize straw. However, the lnA values of *U.pertusa* were higher than that of maize straw which indicated that the pyrolysis process of the former would be easier than the latter. This suggested that co-firing of the two biomass may be require less external heat input and improve process stability. The activation energy distribution of *U.pertusa* were wider than maize straw.

**Table 5:** The activation energies obtained by FWO method and KAS method at different conversion rate of the samples.

Sample	Conversion rate (a)	FWO method			KAS method			Popescu method			
		E^a^ (KJ/mol)	|r^c^|	lnA^b^ (min^−1^)	E (KJ/mol)	|r|	lnA (min^−1^)	Conversion rate (a)	E (KJ/mol)	|r|	lnA (min^−1^)
Maize straw	0.1	132.3	0.9938	22.9	130.1	0.9930	22.4	0.2–0.1	154.7	0.9820	29.5
	0.2	147.9	0.9852	27.5	146.3	0.9834	27.3	0.3–0.2	154.7	0.9715	30.0
	0.3	151.9	0.9780	28.9	150.2	0.9753	28.7	0.4–0.3	158.7	0.9629	30.8
	0.4	155.9	0.9697	29.9	154.2	0.9660	29.7	0.5–0.4	155.2	0.9899	30.3
	0.5	157.8	0.9832	30.5	156.0	0.9811	30.3	0.6–0.5	142.4	0.9896	28.4
	0.6	153.4	0.9871	30	151.2	0.9854	29.6	0.7–0.6	163.2	0.9872	33.1
	0.7	156.9	0.9872	31.2	154.8	0.9855	30.8	0.8–0.7	159.4	0.9637	32.9
	0.8	157.5	0.9813	31.7	155.2	0.9789	31.4	0.9–0.8	163.5	0.9179	34.1
	0.9	157.3	0.9595	32.2	154.8	0.9542	31.9				
	Average	152.3			150.3				156.5		
	Average	153.0									
*Ulva pertusa*	0.1	161.8	0.99966	32.5	161.8	0.99961	32.5	0.2–0.1	157.0	0.99997	33.4
	0.2	159.4	0.99997	33.4	159.1	0.99997	33.3	0.3–0.2	169.3	0.99997	37.7
	0.3	162.6	0.99997	35.2	162.3	0.99996	35.1	0.4–0.3	172.3	0.99997	39.2
	0.4	165.1	0.99997	36.6	164.9	0.99996	36.5	0.5–0.4	155.1	0.99294	35.4
	0.5	161.8	0.99917	36.4	161.4	0.99906	36.2	0.6–0.5	137.8	0.97996	32.0
	0.6	155.4	0.9997	35.3	154.6	0.99968	35.1	0.7–0.6	131.6	0.99982	30.8
	0.7	147.3	0.99997	33.8	146.0	0.99997	33.5	0.8–0.7	123.2	0.93231	29.3
	0.8	137.4	0.99382	32.1	126.8	0.99493	29.4	0.9–0.8	95.0	0.98853	23.4
	0.9	120.3	0.99999	28.6	123.5	0.99983	29.2				
	Average	152.3			151.2				142.7		
	Average	148.7									

a Activation energy;

b Pre-exponential factors;

c Coefficient constant.

Comparisons of the decomposition temperature and activation energy of several types of biomass are provided in Table 6 [1], [2], [13], [14], [15]. The results indicated that the decomposition temperature of algae is lower than that of the higher plants, however the activation energies do differ. These findings suggest that thermal behavior is greatly influenced by feedstock choice.

**Table 6:** Comparison of various kinetic parameters of pyrolysis for different biomass.

Samples	Decomposition temperature (°C)	Activation energy (kJ/mol)	References
Maize straw	200–408	153.0	Present study
*U. pertusa*	182.3–316	148.7	Present study
*Enteromorpha prolifera*	174–551	228.1	[1]
*Laminaria japonica*	192–372	207.7	[2]
*Sargassum pallidum*	172–414	202.9	[2]
Sodium alginate	204–285	188.1	[2]
Rice husk	225–350	79.9	[13]
Cotton stalks	480–630	40.84	[14]
Sunflower shells	300–600	73.81	[15]

Table 7 shows the kinetic compensation effects of the pre-exponential factors and activation energies. In addition to the pre-exponential factors and the activation energy calculated by Popescu methods of *U. pertusa*, there were minor kinetic compensation effects between the pre-exponential factors and the activation energies.

**Table 7:** Kinetic compensation effects of the pre-exponential factors and the activation energy.

Sample	Method	Equation	r
Maize straw	FWO method	lnA = −22.0+0.3E	0.9756
	KAS method	lnA = −22.3+0.3E	0.9732
	Popescu method	lnA = −9.2+0.3E	0.8606
*Ulva pertusa*	FWO method	lnA = 11.9+0.1E	0.8534
	KAS method	lnA = 10.7+0.2E	0.8816
	Popescu method	lnA = 5.5+0.2E	0.9853

### Conclusions

The pyrolysis of biomass can be influenced by the choice of biomass type, pyrolytic temperature, and heating rate. Therefore, the pyrolytic characteristics and kinetics need to be studied for each type of biomass under consideration as feedstock, prior to designing thermal-chemical conversion systems.

Our studies found that there were three stages during the pyrolysis of maize straw and *U. pertusa* which were distinguished as moisture evaporation (stage I), a main pyrolysis process (stage II) and a slow decomposition process (stage III). However, there are significant differences between stages for the different materials. *U. pertusa* is easier to pyrolyse than maize straw. There is more residue from the pyrolysis of *U. pertusa* than from maize straw, indicating that the inorganic salts should be washed out of *U. pertusa* before use as a pyrolysis feedstock.

In addition, there are differences between pyrolysis mechanisms. The primary devolatilization stage of *U. pertusa* can be described by Avramic-Erofeev equation (n = 3), whereas that of maize straw can be described by Mampel Power Law (n = 2).

Further investigation of pyrolysis products is required to fully understand the mechanisms of thermal degradation of the two samples.

## Materials and Methods

### Sample preparation

*U. pertusa* was collected in June of 2009 from the Zhanqiao piers in Qingdao, China. Dry maize straw was collected from the maize planting regions surrounding Liaocheng, Shandong province, China. *U. pertusa* was sun dried for four days, after which both the *U. pertusa* and the dry maize straw were pulverized in a plant disintegrator to all passing a 120 mesh sieve. All samples were stored in a desiccator.

### Proximate analysis of the samples

The moisture analysis was conducted according to established methods [16]. The ash content was determined according to the method described in [17]. The volatile matter content was analyzed according to the method described in [18]. The fixed carbon was expressed as the 100%-ash content-volatile matter-moisture content. Calorific values were determined according to the method described in [19]. All measurements were replicated three times.

### Pyrolysis of the samples

The pyrolytic characteristics were determined according to Li et al. (2010) [2]. Ten milligrams of the sample were put into platinum crucibles with lids on a high accuracy DSC-cp sample holder of a thermal analyzer (TG/DSC STA449, NETZSCH Instruments Co. Ltd., Germany), after which they were heated from ambient temperature to 800°C at rates of 10, 30 and 50°C/min in the furnace under a nitrogen atmosphere of 80 ml/min. The weight loss and calorific changes in response to temperature were then recorded and used to plot the thermogravimetric analysis (TGA), derivative thermogravimetric analysis (DTG) and differential scanning calorimetric (DSC) curves. All experiments were replicated three times.

### The kinetic parameters of the samples

The most probable mechanism was determined by the Popescu method [20], [2], and the activation energy and pre-exponential factor were calculated by using the Popescu method [20], [2], FWO method [21]–[22], [1]–[2] and KAS method [23], [2].

All plots were generated and the lines were fitted using the Origin 7.5 software package (OriginLab Corporation).
